# National Assessment of Surprise Coverage Gaps Provided to Simulated Patients Seeking Emergency Care

**DOI:** 10.1001/jamanetworkopen.2020.6868

**Published:** 2020-05-15

**Authors:** Vivek Parwani, Andrew Ulrich, Craig Rothenberg, Jeremiah Kinsman, Matthew Duhaime, Melissa Thomas, Arjun Venkatesh

**Affiliations:** 1 Department of Emergency Medicine, Yale University School of Medicine, New Haven, Connecticut; 2 Department of Emergency Medicine, Yale New Haven Health System, New Haven, Connecticut; 3 Yale University School of Medicine, New Haven, Connecticut; 4 Center for Outcomes Research and Evaluation, Yale University School of Medicine, New Haven, Connecticut

## Abstract

This cross-sectional study evaluates surprise coverage gaps in health insurance by using secret shopper phone call methods to assess whether hospital billing staff in the US are able to answer a set of patient questions about insurance coverage.

## Introduction

Surprise out-of-network bills, which essentially represent surprise coverage gaps, have recently garnered public outrage and the attention of Congress. These coverage gaps occur when patients seek hospital-based services and incur charges from multiple providers participating in different insurance networks or from out-of-network providers practicing at an in-network hospital. Emergency care has been a central focus of policy makers, as patients have no option to select a provider, and the magnitude of charges can be enormous.^1^

Increasing transparency, especially price transparency, has been heralded as a consumer-based solution to the high price of health care.^2^ However, price transparency does not necessarily imply coverage transparency. Accordingly, we examined surprise coverage gaps from the patient perspective by conducting a national cross-sectional study of hospitals by using secret shopper phone call methods to assess whether patients could receive timely responses to 3 simple insurance coverage questions.

## Methods

This study was considered to be non–human subject research and was exempt from review per Yale Human Research Protection Program guidelines. This study followed the Strengthening the Reporting of Observational Studies in Epidemiology (STROBE) reporting guideline.

For this cross-sectional study, trained research assistants posing as patients seeking emergency care made calls to every acute care hospital in the United States.^3,4^ Calls were conducted from August 1, 2017, to April 30, 2018. Patients stated they were covered by the most subscribed commercial insurance company within their state.^5^ Patients asked hospital billing staff 3 core and 2 follow-up insurance coverage questions (Table). Additional details on the calling procedure are given in the eMethods in the Supplement. We conducted contingency table analyses with χ^2^ testing to compare the probability that patients would receive a separate bill by whether emergency department (ED) physicians were hospital employees. Data were analyzed from July 1, 2018, to March 31, 2020. Statistical tests were 2-tailed, with *P* < .05 representing statistical significance.

**Table.** Responses From Hospitals’ Billing Staff (N = 4231) to Secret Shopper Patient Calls Regarding Surprise Out-of-Network Coverage Gaps^a^

Patient call questions	Yes	No	Unclear^b^	Unanswered^c^
1. Do you take my insurance?	4052 (95.8)	7 (0.2)	149 (3.5)	23 (0.5)
2. Will I get a separate bill from my emergency department doctor?	3147 (74.4)	355 (8.4)	147 (3.5)	585 (13.8)
2a. Will the separate bill be considered in network?^d^	1524 (48.4)	76 (2.4)	1523 (48.4)	24 (0.8)
3. Do your emergency department doctors work for the hospital?	637 (15.1)	2195 (51.9)	240 (5.7)	1159 (27.4)
3a. Who do your doctors work for?^e^	254 (10.4)^f^	NA	177 (7.3)	2004 (82.3)

Abbreviation: NA, not applicable.

^a^ All values are presented as number (percentage).

^b^ Hospital billing staff responded that they did not know the answer to the question.

^c^ In some cases, the initial call taker answered some questions but could not answer all questions. After receiving responses to 1 or 2 questions, simulated patients were placed on hold for extended periods of time, became disconnected, or were transferred and the call went unanswered or to voicemail. Callbacks were not attempted.

^d^ Question 2a was asked if the answer to question 2 was “yes.”

^e^ Question 3a was asked if the answer to question 3 was “no” or was unclear.

^f^ Values indicate the number (percentage) of staff who answered the question.

## Results

Simulated patients connected with hospitals having operational EDs in 4231 (89.0%) of 4752 total calls (Table). In 4059 of 4231 calls (96.0%), the billing staff was able to answer whether the hospital accepted the patient’s insurance. In 2623 calls (62.0%), patients received “yes” or “no” responses to all 3 core questions. Responses received by patients showed that separate professional billing for ED physicians varies by state, with North and South Dakota and several northwestern states having a comparatively higher prevalence of separate billing (Figure). Among 2435 hospitals with billing staff responding that they do not employ their emergency physicians or giving an unclear response, 2092 (85.9%) were unable or unwilling to answer the question of who employed their ED physicians. Responses received by patients showed that hospital employment of ED physicians also varied by state (Figure). The proportion of hospitals reporting employment of ED physicians varied by state from 0% (Indiana, Kentucky, Missouri, Oklahoma, Tennessee, Utah, and Wisconsin) to 64.2% (North Dakota), with a mean (SD) of 20.0% (18.1%) reporting direct employment of emergency physicians. Our analysis showed that the probability of receiving a single professional and hospital bill for emergency care was associated with hospital employment of ED physicians (*r *= 0.49;* P < *.001).

**Figure.**
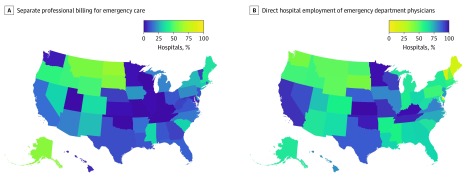
State-by-State Prevalence of Separate Professional Billing for Emergency Care and Direct Hospital Employment of Emergency Department Physicians A, Map shows the percentage of acute care hospitals’ billing staff responding to secret shoppers that they would receive a separate bill from the emergency department doctor. B, Map shows the percentage of acute care hospitals’ billing staff responding to secret shoppers that their emergency department doctors work for the hospital.

## Discussion

In this cross-sectional study of more than 4000 hospitals, we were reassured to find that 96.0% of hospitals’ billing staff could quickly answer whether they accepted the patient’s insurance. However, only 62.0% gave immediate “yes” or “no” responses to all 3 core questions. Furthermore, nearly half of those informing patients they would receive a separate professional bill from the emergency physician could not answer whether the bill would be considered in network. Also concerning, nearly one-third of billing staff were unable or unwilling to answer whether their ED physicians were hospital employees. These findings suggest that the current system cannot accommodate the coverage information needs of many patients seeking emergency care.

Despite national efforts to increase price transparency and evidence of price transparency successes for nonemergent and scheduled care, our results illustrate the limitations of transparency efforts in solving a surprise coverage gap problem.^6^ Specifically, the percentage of unclear and unanswered responses to questions 2, 2a, and 3 demonstrates the practical obstacles patients face when trying to quickly determine insurance coverage for emergency care. Furthermore, the observed geographic variation suggests that a single transparency solution is unlikely to universally address this issue.

As Congress debates legislation to address surprise billing, we propose that plans (ie, employer based, exchange based, Medicare, and Medicaid) eliminate out-of-network penalties and prior authorization requirements for emergency care and provide standardized, available pricing that applies equivalently to all. This approach does not assume zero out-of-pocket spending for patients, but it would eliminate surprise coverage gaps for emergency care and allow for predictable and reasonable billing. This study’s limitations include the following: (1) the unique study questions have not been validated in the research literature, and (2) the accuracy of the responses received were not validated.

## Notes

Supplement. **eMethods.** Secret Shopper Calling Procedure
