# Supplementary material for: National Assessment of Surprise Coverage Gaps Provided to Simulated Patients Seeking Emergency Care
Source: JAMA Netw Open. 2020 May 15;3(5):e206868. doi: 10.1001/jamanetworkopen.2020.6868 (PMC7229522; doi:10.1001/jamanetworkopen.2020.6868)
Supplement: Supplement. — eMethods. Secret Shopper Calling Procedure [file jamanetwopen-3-e206868-s001.pdf]

Supplementary Online Content

Parwani V, Ulrich A, Rothenberg C, et al. National assessment of surprise coverage gaps provided to simulated patients seeking emergency care. *JAMA Netw Open*. 2020;3(5):e206868. doi:10.1001/jamanetworkopen.2020.6868

eMethods. Secret Shopper Calling Procedure

This supplementary material has been provided by the authors to give readers additional information about their work.

© 2020 Parwani et al. *JAMA Network Open.*

eMethods. Secret Shopper Calling Procedure

The three core questions and two follow-up questions were developed by the study team. The questions were pilot tested locally on hospital employees not participating in the study and then iteratively improved after the first 10 calls to ensure understandability and clarity. Calls were made to the main hospital number listed on the CMS Hospital Compare website. If there was no answer to a listed number, callers "Googled" the hospital name and called the main number listed. Callers asked to speak with hospital billing staff. After being transferred, callers confirmed that they were speaking with someone who could answer emergency care insurance coverage and billing questions. When call takers could not answer a question, callers asked if there was someone else who could answer the question. When all call takers responded that they did not know the answer to a question, responses were coded as *unclear*. When transferred calls were disconnected or went to voicemail, responses were coded as *unanswered*. Callbacks were not attempted.

© 2020 Parwani et al. *JAMA Network Open.*
